# Supplementary material for: The Evolution and Characterization of the RNA Interference Pathways in Lophotrochozoa
Source: Genome Biol Evol. 2024 May 7;16(5):evae098. doi: 10.1093/gbe/evae098 (PMC11114477; doi:10.1093/gbe/evae098)
Supplement: evae098_Supplementary_Data [file evae098_supplementary_data.zip › Supplementary_materials.zip!/selected_miRNAs/NoGe_selected_miRNAs/known_miRNA_families_pdf_files/NMRB01000152.1_Notospermus_geniculatus_strain_Ushimado_scaffold152,_whole_genome_shotgun_sequence_5839.pdf]

Provisional ID	:	NMRB01000152.1_Notospermus_geniculatus_strain_Ushimado_scaffold152,_whole_genome_shotgun_sequence_5839
Score total	:	17140
Score for star read(s)	:	3.9
Score for read counts	:	17130.8
Score for mfe	:	2.3
Score for randfold	:	0
Score for cons. seed	:	3
Total read count	:	33613
Mature read count	:	33528
Loop read count	:	0
Star read count	:	85

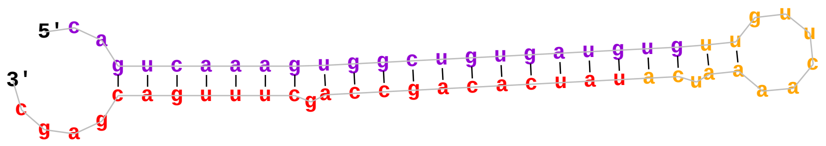

Genome coordinates: NMRB01000152.1 415241-415299

pre-miRNA sequence:
CAGUCAAAGUGGCUGUGAUGUGCAGUCAAAGUGGCUGUGAUGUGUUGUUCAAAAUCAUAUCACAGCCAGCUUUGACGAGC

Mature sequence: UAUCACAGCCAGCUUUGACGAGC

Aligned mature sequence of the *N. geniculatus* pre-miRNA with the top 5 hits from the BLAST search against the pre-miRNA mirGeneDB set:

```
                                ********** ******* ***
    Nge_Mir-2-P4i               uaucacagccagcuuugacgagc-
    Lpo-Mir-2-P3h_3p            uaucacagccagcuuugaugagca
    Cgi-Mir-2-o30_3p            uaucacagccagcuuugaugagcu
    Csc-Mir-2-P4o_3p            uaucacagccagcuuugaugagcg
    Lan-Mir-2-o73_3p            uaucacagccugcuuugaugaga-
    Lpo-Mir-2-P4i_3p            uaucacagccagcuuugaugagcg
```
